# Supplementary figures and images for: Presynaptic inhibition rapidly stabilises recurrent excitation in the face of plasticity
Source: PLoS Comput Biol. 2020 Aug 7;16(8):e1008118. doi: 10.1371/journal.pcbi.1008118 (PMC7439813; doi:10.1371/journal.pcbi.1008118)

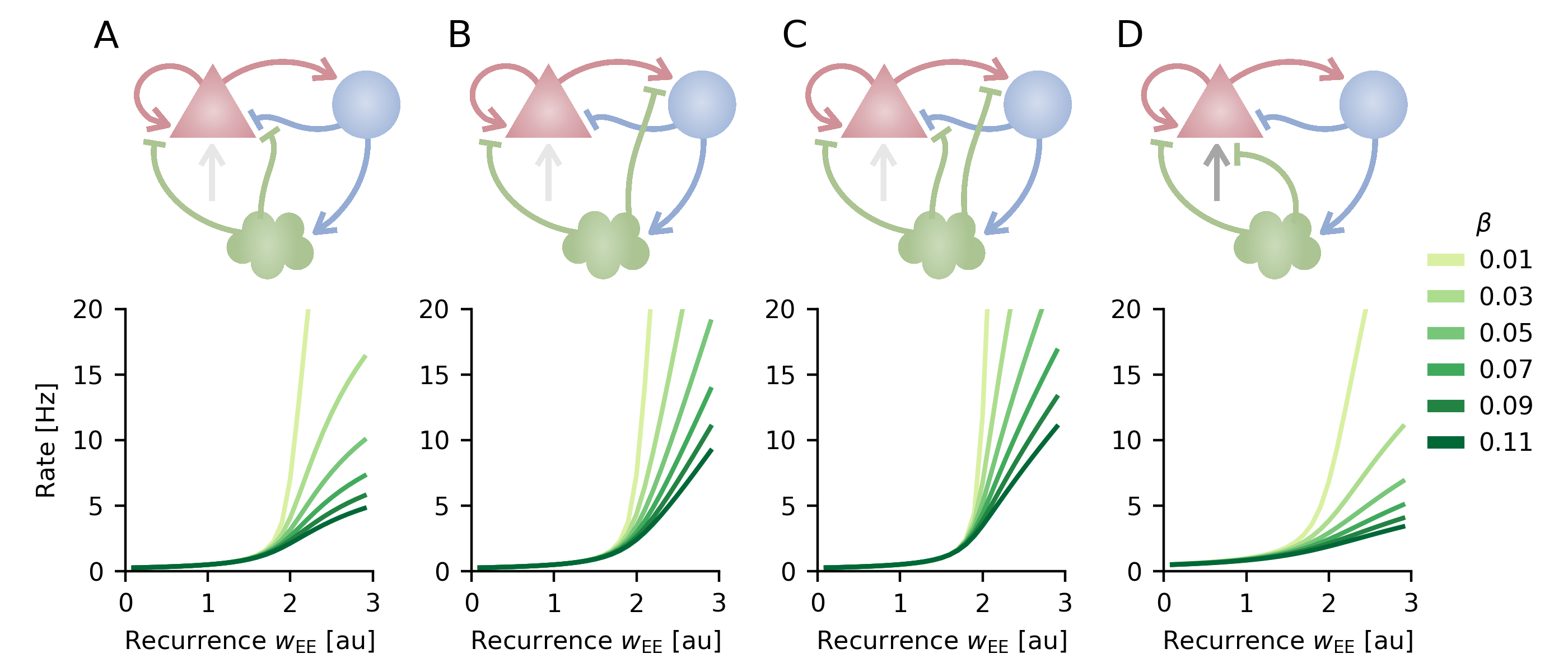

Supplement: S1 Fig — Top: Mean population models in which presynaptic inhibition not only affects excitatory recurrent synapses, but also A. inhibitory synapses onto excitatory cells, B. excitatory synapses onto inhibitory cells C., all recurrent synapses (including recurrent inhibitory connections) or D. background input. Bottom: Firing rate of excitatory population in respective circuit model as a function of excitatory recurrence for increasing strengths of presynaptic inhibition (β). (TIF) [file pcbi.1008118.s001.tif]

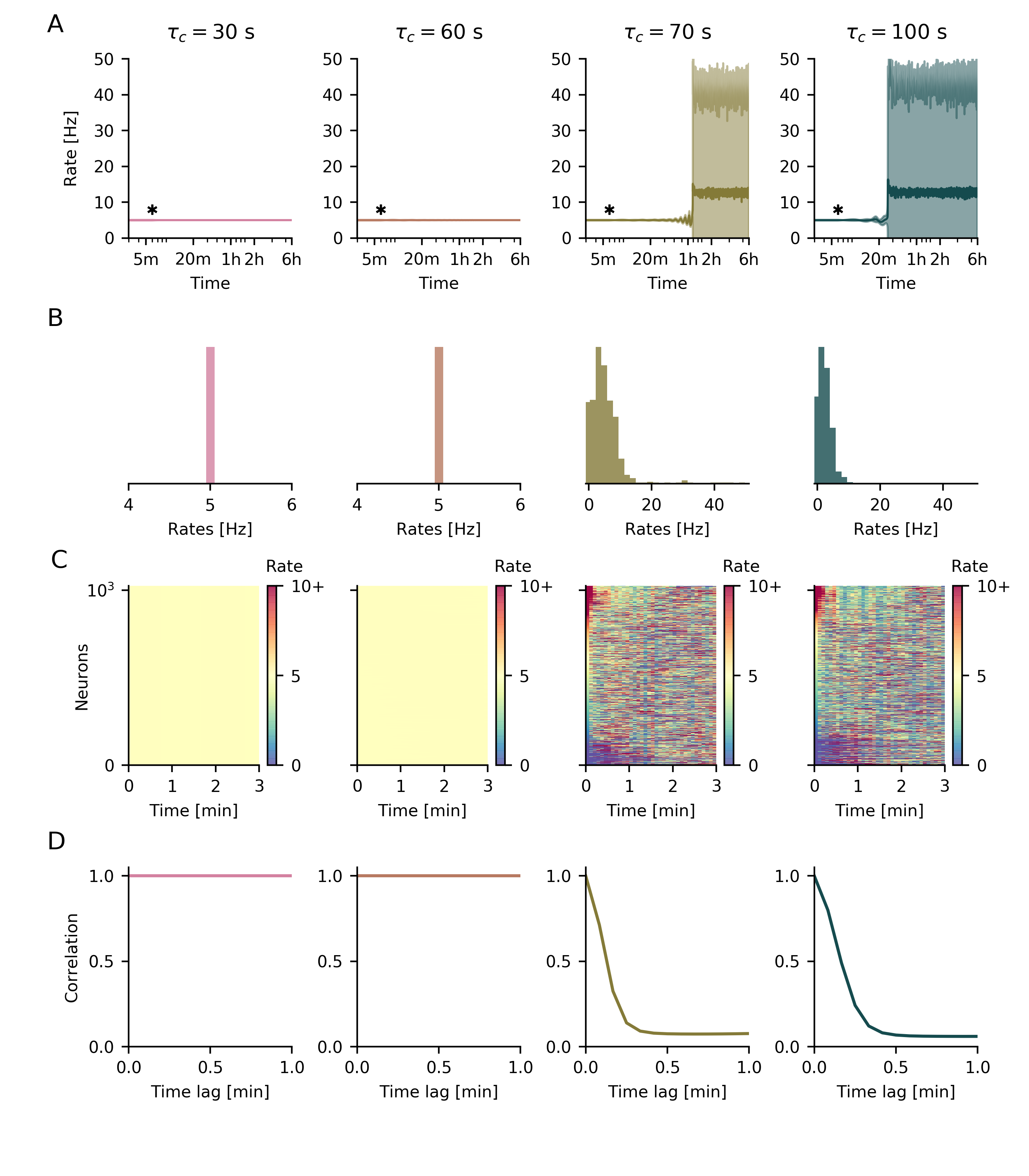

Supplement: S2 Fig — Fast homeostasis (τc = 30 s) produces a narrow distribution around the target rate of 5 Hz. Slower homeostasis leads to a broad long-tailed distribution of firing rates with an inherent turnover but no temporal oscillations. A. Mean (solid line) and standard deviation (shaded area) of firing rate over time. Plasticity is switched on after 6 minutes of simulation time (asterisk). B. Distribution of single neuron firing rates. C. Firing rate of single neurons over time. Target rate is indicated by yellow colour. D. Temporal correlation of single neuron firing rates. (TIF) [file pcbi.1008118.s002.tif]

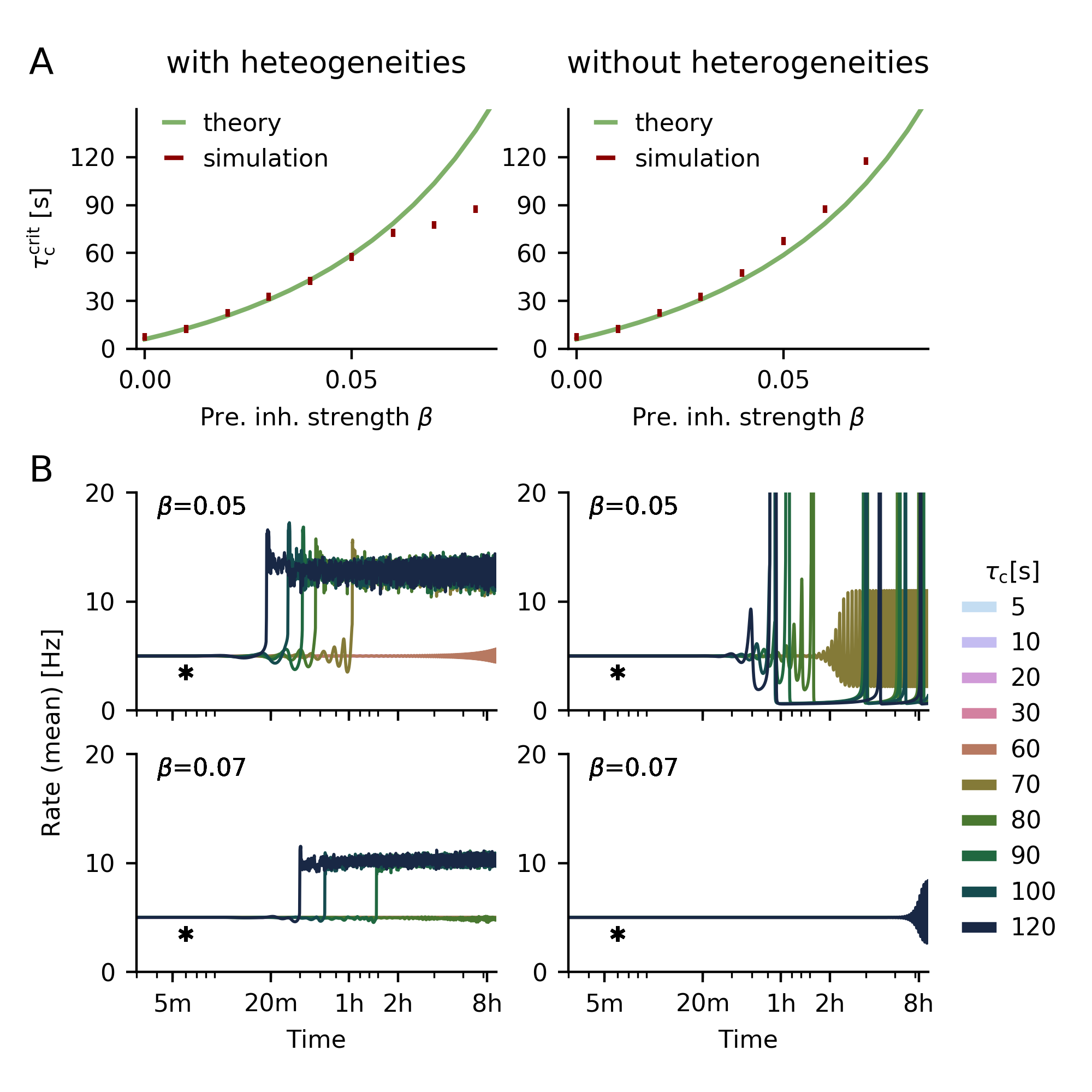

Supplement: S3 Fig — With heterogeneity in the initial excitatory recurrent weights, the theory holds as long as presynaptic inhibition is not too strong. Removing this source of heterogeneity from the network increases the critical timescale for strong presynaptic inhibition, and thereby provides a better match to the analytically determined critical timescales. A. Critical homeostatic time scale as a function of the presynaptic inhibition strength β in networks with (left) and without (right) heterogeneity. For simulations the vertical bars indicate the range between largest stable and smallest unstable timescale tested. B. Temporal evolution of the average firing rate for different homeostatic time constants τc with (left) and without (right) heterogeneity in the network for different strengths of presynaptic inhibition β. (TIF) [file pcbi.1008118.s003.tif]

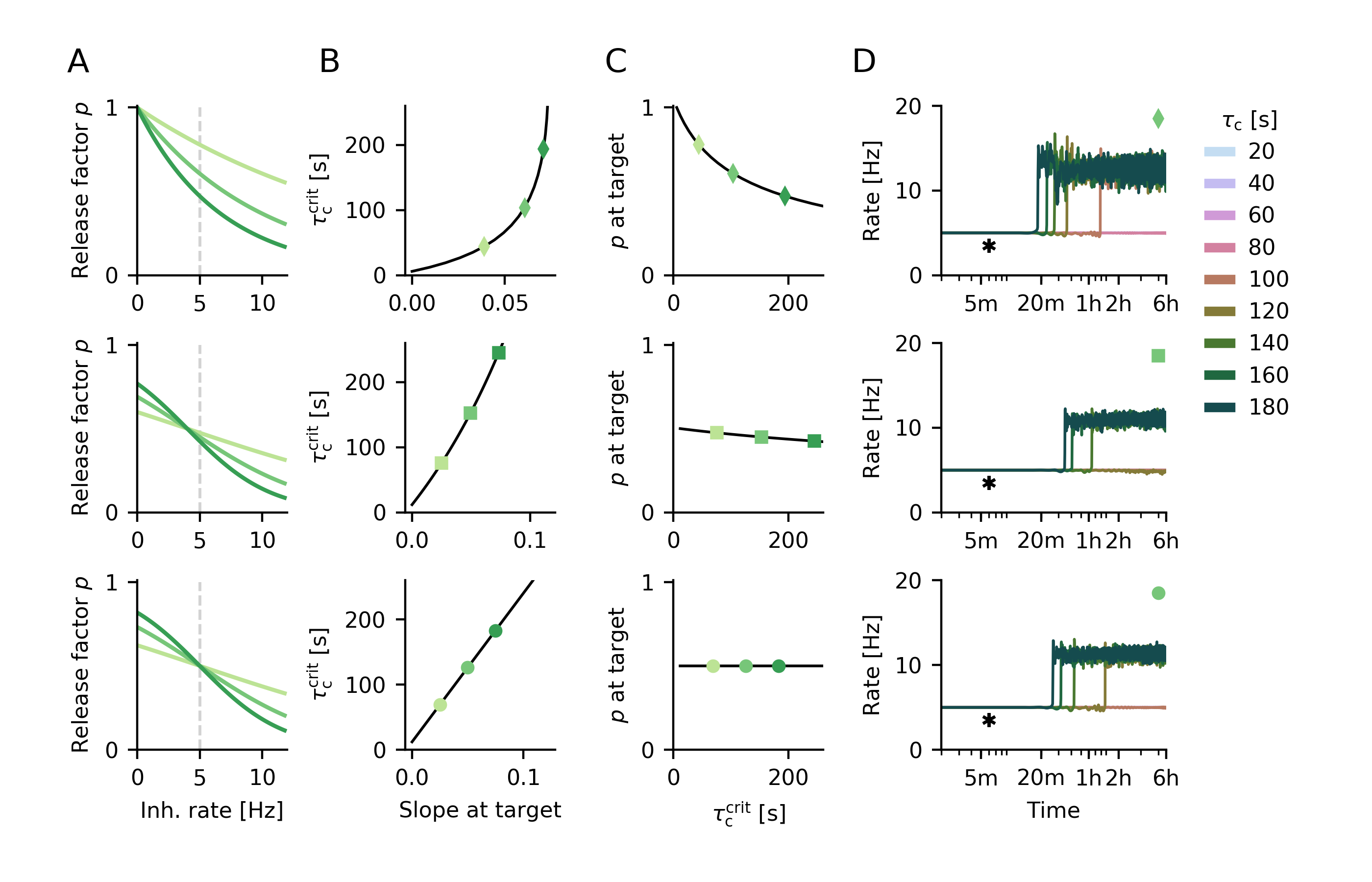

Supplement: S4 Fig — We qualitatively recover the results obtained for linear transfer functions if the release probability decreases exponentially or as a sigmoid with increasing inhibitory rate. A. Exponentially decreasing (top) and sigmoid transfer functions (bottom two) linking inhibitory firing rate to release factor for different slope parameters (βe = 0.05, 0.1, 0.15, βs = 0.1, 0.2, 0.3, shift of sigmoid: 4 (middle) and 5 (bottom). Target rate of 5 Hz is indicated by gray dashed line. B. Increase in analytical critical homeostatic timescale as a function of transfer function slope at the target rate. Parametrisation in A indicated by markers of same colour. C. Release factor at target rate as a function of increase in homeostatic time constant. Markers correspond to transfer functions shown in A. D. Temporal evolution of the average firing rate in networks with presynaptic inhibition for different homeostatic time constants τc. Plasticity is switched on after 6 minutes of simulation time (asterisk). Coloured marker in top right corner specifies parametrisation of transfer function (see A). (TIF) [file pcbi.1008118.s004.tif]

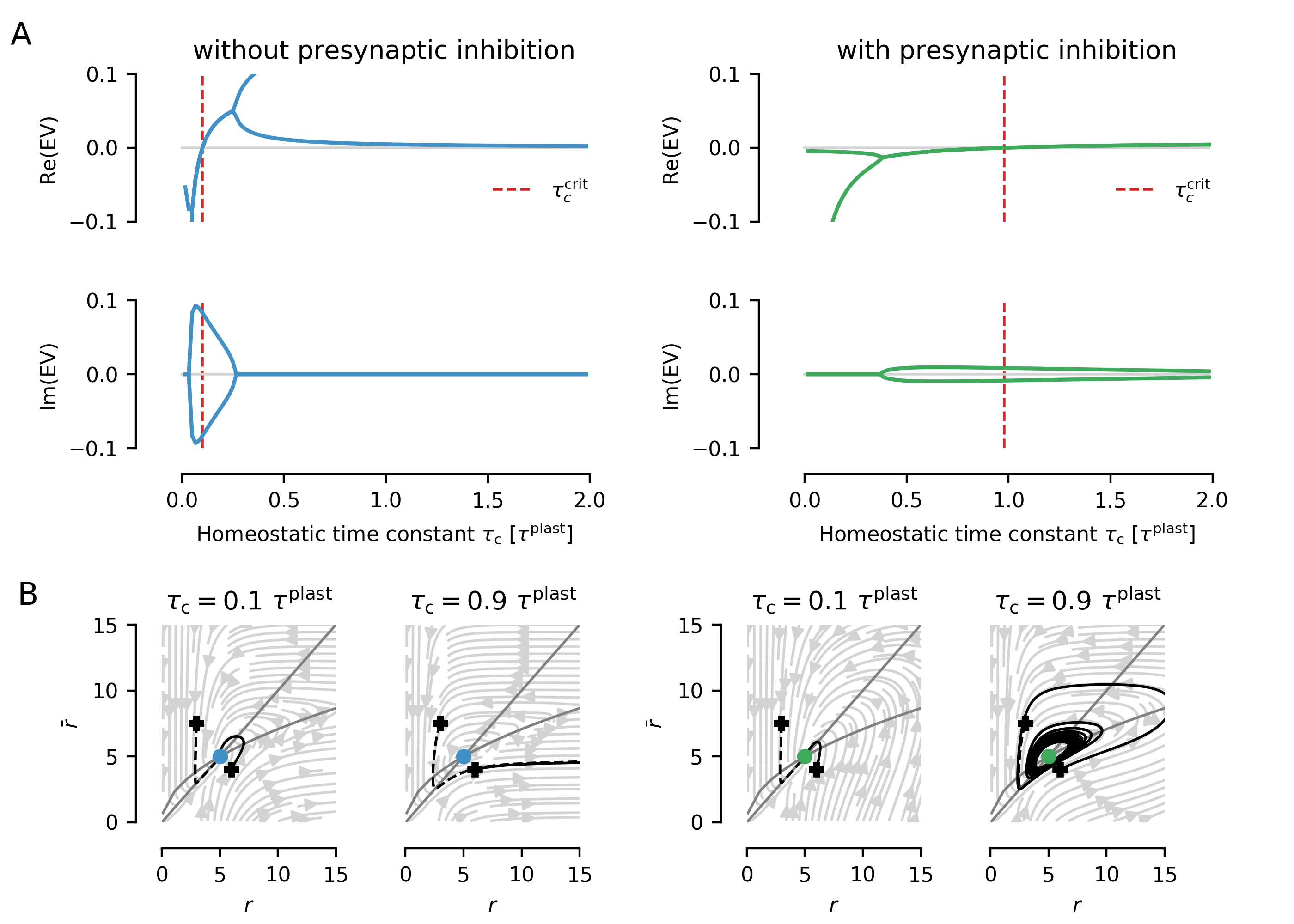

Supplement: S5 Fig — Besides increasing the critical homeostatic time constant, presynaptic inhibition preserves low firing rates beyond the point of stability. Systems without presynaptic inhibition trajectories diverge away from the fixed point (i.e. target rate, 5 Hz) if homeostasis is close to the timescale of plasticity, whereas systems with presynaptic inhibition merely oscillate around it. A. Eigenvalues of reduced system with compared to without presynaptic inhibition as a function of homeostatic time constant. The time constant is given in units of effective plasticity time constant τplast. The critical homeostatic timescale (where the eigenvalues become positive) is marked by a red dashed line. B. Phase plane dynamics with nullclines and example trajectories for short and long homeostatic timescales in systems with and without presynaptic inhibition. (TIF) [file pcbi.1008118.s005.tif]
